# From Childhood Burdens to Relationship Strains: Exploring Partner Parentification and Couple Burnout

**DOI:** 10.3390/bs16020177

**Published:** 2026-01-26

**Authors:** S. Burcu Özgülük Üçok, Gizem Öztemür, Özge Yılancı

**Affiliations:** 1 Department of Guidance and Psychological Counseling, TED University, Ankara 06420, Türkiye; ozge.yilanci@tedu.edu.tr; 2 Independent Researcher, Balıkesir 10100, Türkiye; gizem.oztemur1@gmail.com

**Keywords:** partner parentification, parentification, couple burnout, multi-method design, marriage

## Abstract

Parentification is the process in which children assume caregiving roles typically designated for parents. Prior research suggests that adults with a history of childhood parentification tend to find themselves in relationships where they continue their caregiving roles, leading to emotional exhaustion and couple burnout. This study examines the associations among childhood parentification, partner parentification, and couple burnout, and investigates the mediating role of partner parentification in the relationship between childhood parentification and couple burnout. A multi-method research design, with a quantitative sample comprising 283 married individuals aged 18–65 who had been married for at least 6 months, was used. The quantitative measures were the Partner Parentification scale, Parentified Child Scale-Adult Version (PCS-A), and Couple Burnout Measure-Short Form (CBMS). Within the scope of the quantitative study, the path analyses revealed that partner parentification partially mediated the association between childhood parentification and couple burnout. Qualitative data were collected to assess partner parentification experiences, and the deductive content analysis results revealed aspects similar to those of childhood parentification. The findings suggest that childhood parentification shares similar aspects with partner parentification, and individuals who were parentified by their parents were eager to be parentified by their spouses, experiencing couple burnout in their relationships.

## 1. Introduction

Family therapists characterize healthy familial interactions as a well-defined hierarchy in which the parent provides leadership and care for the child, where the child seeks comfort and guidance from the parent (Minuchin, 1974). However, upon disruption of this hierarchical structure, children may assume parental duties when a parent relies on them for support rather than seeking assistance from another adult (Jacobvitz et al., 1999). This phenomenon was initially conceptualized as parentification by Boszormenyi-Nagy and Spark (1973), denoting role reversal within the family system. In essence, this term underscores a blurred hierarchy or breakdown of boundaries in family dynamics, whereby children are excessively compelled to assume emotional (such as caring, supporting, and guiding) and instrumental (such as cooking, cleaning, and managing finances) roles that are traditionally designated for adults (Hooper, 2007; Kerig, 2005; Nuttall & Valentino, 2017). The definition of parentification, therefore, differs from parental supervision or monitoring of a child’s household tasks or responsibilities, which parents assign to their child to promote their development. According to Hooper (2011, 2013), the central theme of parentification is that the parentified child or youth assumes the role of caregiver, providing care for others (i.e., parents, younger siblings) at the expense of their own care. Thus, a parentified child typically assumes parental roles toward their own parents and siblings, while also having to parent themselves. The process of parentification, therefore, involves the allocation of an excessive degree of responsibility and authority to them with respect to themselves and other members of the family.

Several factors have been identified as risk factors for the parentification of children, including divorced or single-parent households, migration, parents’ excessive reliance or dependence on alcohol or other substances, mental health issues in parents, lower socio-economic status, family stress, the presence of a child with a disability or chronic illness, and family conflict (e.g., Byng-Hall, 2008; Dariotis et al., 2023; Kerig, 2005; Masiran et al., 2023; Nuttall et al., 2018). Furthermore, several terms have emerged in current research to describe these dynamics, emphasizing the nuanced nature of this relational shift. For example, it is often referred to as childhood parentification (i.e., Madden & Shaffer, 2016; Masiran et al., 2023), the parental or parentified child (i.e., Chase, 1999), retrospective reports of parentification (i.e., Baggett et al., 2013; Jurkovic et al., 2001), parentification during childhood (i.e., Tolmacz et al., 2025), and childhood adultification (i.e., Burton, 2007).

Given that parentification is characterized by ambiguous boundaries among family members, it is essential to explore how this phenomenon manifests within the relationships typical of the Turkish family system. The Turkish family system is often characterized by highly interdependent relationships, with children playing a material and psychological value in the family structure (Kağıtçıbaşı & Sunar, 1992). For instance, children in Türkiye anticipate supporting their families financially by contributing to household income upon commencing their careers, assisting with their siblings’ educational expenses, and providing care and financial support to their parents in old age (Kağıtçıbaşı, 1993). Nevertheless, socioeconomic developments within the country have led to a reduction in material interdependencies across generations. At the same time, psychological (emotional) reliance has not only persisted but also become more pronounced, underscoring emotional interdependence (Kagitcibasi & Ataca, 2005). This change indicates that although the nature of intergenerational dependence may have shifted from primarily instrumental to more emotional, a strong sense of interdependence among family members and uncertainty about family boundaries remain key features, creating a context well-suited for parentification.

The majority of studies conducted to date (over three-quarters) have concentrated on the detrimental effects of parentification (see Dariotis et al., 2023), while some research acknowledges potential positive outcomes, such as enhanced resilience, social skills, or improved sibling relationships (Boumans & Dorant, 2018; Burton, 2007; Masiran et al., 2023). Nevertheless, the adverse effects of parentification encompass a spectrum of issues, including the inability to develop a distinct psychological identity due to the dissolution of boundaries within the family system (Kerig, 2005), as well as diminished self-esteem and reduced self-worth, whereby individuals often evaluate themselves as less attractive or capable owing to premature burdens they bear (Black & Sleigh, 2013). This focus is well-founded, given that the roles and responsibilities allocated to the child in such contexts frequently exceed what is developmentally, emotionally, and age-appropriately expected (Hooper et al., 2014; Nuttall & Valentino, 2017). The subjective experiences of these children often reflect these systemic issues; many perceive their obligatory adult roles as unjust and as a deprivation of their childhood, resulting in substantial stress, role overload, and resentment. Furthermore, parentification may impede children’s opportunities to learn and adopt positive coping mechanisms typically transmitted through parental guidance. As previously documented, the adverse consequences of these experiences include a broad array of psychological issues, such as distress, depression, interpersonal difficulties, and a tendency to view oneself as a burden (Hooper et al., 2011a, 2011b; Masiran et al., 2023).

The long-term consequences of childhood parentification could extend into adult romantic partnerships, where attachment theory provides a robust conceptual framework for understanding how these early caregiving dynamics shape later relational patterns, as childhood parentification can significantly influence children’s attachment to their parents (e.g., Hooper, 2007). From an attachment perspective, children and adolescents raised in unsupportive or unmotivating homes, or lacking a consistent attachment figure or secure base who is available, responsive, and helpful when needed, often struggle to form close, intimate relationships (Bowlby, 1989). Parentification frequently involves the dissolution of generational boundaries and role reversals, which are correlated with insecure attachment styles within familial relationships. Consequently, these initial disruptions in attachment are believed to influence subsequent social interactions, as insecure attachment established during childhood may endure into adult relationships (see Byng-Hall, 2008). For example, Madden and Shaffer (2016) found a positive link between reports of parentification in childhood and difficulties in romantic relationships, including reduced ability to communicate effectively and a higher likelihood of anxious attachment. Researchers have also identified a strong connection between childhood parentification and detrimental communication patterns in adult romantic partnerships. This can lead to anxious attachment-related thoughts, such as jealousy and deep uncertainty about a partner's love. These dynamics are significant because they impact the well-being of both partners and shape their relationship as a whole. Additional studies have similarly shown a negative correlation between childhood parentification and marital satisfaction (Baggett et al., 2013; Zencir, 2021).

Similarly, in their recent study, Tolmacz et al. (2025) found that people who perceived themselves as parentified during childhood (taking on parental roles as children) reported lower relationship satisfaction with their romantic partners, due to greater experiences of unmet psychological needs. Researchers also noted that adults with retrospective accounts of parentification during childhood may struggle to recognize mutuality in future relationships, as they often repeat similar cycles and continue to be parentified by their partners in current romantic relationships. In sum, this situation could pose a risk to future healthier relationships, characterized by healthy boundaries (i.e., mutual giving and taking) in family dynamics among adults (Tolmacz et al., 2025).

Previous studies (Emre, 2016; Engelhardt, 2012) highlight the need for further research to deepen understanding of the relationship between childhood parentification and its impact on future adult relationships by examining the nature of this relationship. While it is known that being parentified during childhood can negatively influence romantic relationships (see Madden & Shaffer, 2016; Tolmacz et al., 2025), the degree to which individuals see themselves as being parentified (by their partner) in their romantic relationships, and the specific situations where they assume a parental role, remains underexplored in current research. Therefore, this study aims to explore how perceived partner-parentification affects the relationship between childhood parentification and couple burnout, as well as to identify the experiences that lead individuals to perceive themselves as taking on a parental role in their partner relationships.

### 1.1. Couple Burnout: Emotional Depletion and the Struggle for Connection

Burnout occurs when a person experiences energy depletion and feels overwhelmed by multiple problems (Freudenberger, 1986), resulting in physical, emotional, and mental exhaustion (Pines & Aronson, 1988). Originally derived from occupational environments, burnout in couples refers to the physical, emotional, and mental exhaustion that arises from sustained involvement in situations that place significant demands on romantic relationships, either instrumental or emotional (Pines, 1987, 1996). According to Pines (1996), burnout in romantic relationships is characterized by a gradual depletion of emotional energy, resulting in feelings of hopelessness and resentment toward one’s partner. This phenomenon transcends mere dissatisfaction; it reflects a profound and pervasive sense of disconnection, as evident in occupational environments, which can significantly affect relationship quality.

In addition to the definition of couple burnout, the literature offers various alternative terms that capture the essence of this challenging experience. These terms include marriage burnout (e.g., Pines, 1987), marital burnout (e.g., Kebritchi & Mohammadkhani, 2016; Jafari et al., 2021), spouse burnout (e.g., Ekberg et al., 1986), and spousal burnout (e.g., Yıldırım & Menteş, 2024). Recognizing these terms is essential because they can illuminate the subtle aspects of emotional fatigue that couples experience, ultimately hindering their ability to maintain healthier relationships.

### 1.2. Childhood Parentification and Couple Burnout

Previous research has shown that the degree of parentification a child experiences may affect their future adult romantic relationships in numerous ways. This phenomenon can lead to various relational patterns, including unhealthy communication styles and attachment issues (Madden & Shaffer, 2016), difficulties with intimacy and relationship satisfaction (Baggett et al., 2013; Zencir, 2021), and challenges in establishing healthy boundaries (Tolmacz et al., 2025). Furthermore, in their qualitative study, Schorr and Goldner (2023) illustrated that childhood parentification can be considered a significant form of relational trauma. The researchers discussed that those who parentified during childhood often perceive their parental figure as threatening (with fear and threat being dominant emotions), neglecting, invalidating their existence as a separate person, and therefore confusing. Acknowledging this dynamic is essential because it can deepen understanding of the complexities involved and help identify potential difficulties that may arise in future close adult relationships.

Similarly, these earlier experiences of parentification may be related to maladaptive schemas in later relationships, which are critical to understanding couple burnout. For instance, Kebritchi and Mohammadkhani (2016) found a negative association between some distorted beliefs and burnout levels in marriage. For instance, many individuals feel that their fundamental emotional needs are not being adequately met by those closest to them. Similarly, Hooper (2007) described parentification as a complex phenomenon akin to emotional neglect. One can infer from these results that when individuals anticipate that their needs will remain unfulfilled in a relationship, they may be reluctant to express those needs openly. Therefore, with this hesitation in mind, one may fear rejection if they express their needs, believing that their desires or needs are unworthy and that others will not consider them. When these needs remain unexpressed and therefore unfulfilled, satisfaction in romantic relationships may decline, which, in some situations, can lead to distance and disconnection (Pines, 1996). Over time, this prolonged neglect may contribute to what is often described as couple burnout, where partners experience extreme exhaustion and disengagement from one another, struggling to revive the intimacy and joy that once defined their bond.

### 1.3. Partner Parentification and Couple Burnout

The existing literature on parentification and its effects on future relationships has primarily focused on retrospective accounts of parentification during childhood and its impact on current romantic relationships (e.g., Baggett et al., 2013; Tolmacz et al., 2025). However, the current literature lacks studies examining how one’s sense of being parentified by a partner in a current romantic relationship (partner parentification) affects the couple’s relationship. Further, to the researchers’ knowledge, no previous studies have examined experiences or perceptions of partner parentification, which is hereby initially mentioned in this manuscript.

According to the existing conceptualization of parentification, one critical determinant is the blurred hierarchy and dissolution of boundaries, which allows children to assume adult responsibilities. Although there is no hierarchy between two adult partners, an equal division of responsibilities and work is often considered ideal in romantic relationships (see Carlson et al., 2016). As Sprecher (1986) discussed, couples frequently exchange resources, responsibilities, love, and affection in their romantic relationships. However, when this exchange is not balanced, couples are likely to experience negative emotions in their marriages (Waddell et al., 2021). For instance, Carlson et al. (2020) and Carlson (2022) found that as the number of equally shared tasks in couples increases, their relationship satisfaction increases. These studies examined whether an egalitarian or equitable distribution of household tasks would be the most mutually advantageous arrangement for couples, thereby yielding the highest levels of relationship quality for both men and women. Similarly, Carlson et al. (2016) found that as couples share equal responsibility for childcare tasks, their relationship satisfaction also increases.

In a similar vein, not only equality in instrumental means but equality in emotional work, which is defined roughly as being empathetic and making active efforts to understand the other party (England & Farkas, 1986), is found to be critical for relational well-being (Pedersen et al., 2011; Strazdins & Broom, 2004). Overall, being parentified by a partner in a relationship can be understood as a situation in which responsibilities (i.e., instrumental and emotional) are unevenly distributed between the two individuals. In these dynamics, one partner often assumes a parental role, which may involve taking on duties that exceed their fair share of responsibilities. In contrast, the other partner may become more passive or reliant. This imbalance can disrupt the mutuality and equality that are vital to a healthy relationship, thereby reducing relationship satisfaction. Ultimately, this situation may increase the likelihood of couple burnout.

### 1.4. The Current Study

This study aims to investigate the relationships between reports of childhood parentification and their association with couple burnout in adulthood, as well as reports of partner parentification. By investigating these associations, the ultimate aim is to uncover how early family dynamics and the levels of sense of being parentified by a partner contribute to one’s level of couple burnout in adult romantic relationships. We also aim to investigate the various ways in which participants perceive their partners as having parentified them, meaning that they feel an excessive level of responsibility or caretaking, typically expected of a parent. As noted above, to our knowledge, no prior studies have examined how individuals perceive themselves as assuming a parental role toward their partners in couple relationships.

This exploration could provide a more nuanced and comprehensive understanding of the phenomenon of parentification. Although parentification literature suggests how individuals’ retrospective accounts of parentification during childhood or adolescent years could contribute to their future well-being in both individual and relational terms, there is no previous study that examines how childhood parentification shows similarities in adult romantic relationships (i.e., emotional and instrumental excessive roles that could also be taken care of by the other adult in the relationship). By adopting a multi-method approach, personal experiences were qualitatively assessed to shed light on similarities and differences (if any) between childhood parentification and partner parentification.

Following the existing literature, two hypotheses and one research question were formed:

**Hypothesis 1 (H1).**  *The reports of childhood parentification will be positively associated with couple burnout levels.*

**Hypothesis 2 (H2).**  *The reports of partner parentification will mediate the relationship between the reports of childhood parentification and couple burnout levels.*

Research question (RQ1): In what ways do individuals sense that they are taking on a parental role toward their partners?

## 2. Materials and Methods

This study employed a multi-method research design, comprising a series of related research projects that focused on a broad theme to address a comprehensive research problem (Brewer & Hunter, 2006). The primary advantage of multi-method research is that it provides a more convincing, inclusive, and flexible approach to communicating scientific ideas (Hunter & Brewer, 2016). Unlike mixed-methods research, in this design each study operates independently and thoroughly on its own, but all together targets the same research problem. Importantly, data from separate studies are usually not combined, whereas data integration is a key aspect of mixed-methods designs (Hankivsky & Grace, 2016). Therefore, this study adopted a multi-method research design, aligning with its objectives.

### 2.1. Quantitative Study

#### Participants and Procedure

The sample of this study comprised participants who met the following recruitment criteria: (1) being older than 18 years old, (2) being married for at least six months, and (3) residing in Türkiye. Although partner parentification can occur in dating relationships, we restricted the sample to couples who had been married for at least six months for both conceptual and methodological reasons. Parentification in romantic relationships typically reflects a chronic imbalance in the distribution of instrumental and emotional responsibilities. Such imbalances become more stable and measurable in marital relationships, where partners are more likely to share daily routines, household tasks, and long-term relational commitments. Hence, the study focused on married individuals who have been married for at least 6 months.

The participants were reached via purposive and snowball sampling. A power analysis (Faul et al., 2009) for χ^2^ tests indicated that the minimum sample size to yield a statistical power of at least 0.95 with an alpha of 0.05 and a medium effect size (d = 0.3) was 220. The current study sample exceeded this size, consisting of 283 married individuals, of whom 179 were women (63.3%) and 104 were men (36.7%). The ages of 80 participants (28.3%) were between 18 and 29, 127 participants (44.9%) were between 30 and 45, and 76 participants (26.9%) were between 45 and 65. The majority of participants (*n* = 161; 56.9%) held an undergraduate degree, and 61 (21.6%) had a graduate degree. It can be inferred that the majority of the sample comprised highly educated participants. When the income levels of the participants were considered, 37 (13.1%) of the participants reported not having any income, 26 (9.2%) had an income between 20,001 TL-30,000 TL, 32 (11.3%) had an income between 30,001 TL-40,000 TL, 51 (18%) between 40,001 TL-50,000 TL, and the majority had an income over 50,000 TL (*n* = 111; 39.2%). Most of the participants had been married for 1–5 years (*n* = 63; 22.3%) or 10–15 years (*n* = 63; 22.3%). The marital duration of the participants was generally distributed homogeneously, as depicted in Table 1.

Before commencing the data collection, approval was obtained from the Human Subjects Ethics Committee of the university affiliated with the first and third authors. Following ethical approval, the data collection instruments were uploaded to Google Forms, and a study announcement was prepared. The online survey, accompanied by the announcement, was then shared on various social media platforms, including LinkedIn, Instagram, Facebook, X, and Telegram. Additionally, a QR code for the online survey was well-prepared and shared with married individuals based on convenience and through snowball sampling. Participants’ voluntariness was ensured via the informed consent presented at the beginning of the online survey. Completing the study took approximately 10 to 15 min. Data collection commenced in early June 2024 and continued until January 2025.

### 2.2. Qualitative Study

#### Participants and Procedure

The participants in the qualitative part consisted of those who were also involved in the quantitative part. The sample consisted of 93 participants, of whom 84 (90%) were women and 9 (10%) were men. The ages of 34 participants (37%) were between 18 and 29 years old, 45 of them (48%) were between 30 and 45 years old, and 14 of them (15%) were between 45 and 65 years old. Similarly to the quantitative part, the sample consisted mostly of highly educated participants, with the majority (*n* = 50; 54%) holding an undergraduate degree, and 25 (27%) holding a graduate degree. In terms of reported income, 12 participants (13%) indicated not having any income, 8 of them reported an income level equal to minimum wage or below minimum wage, 3 of them (0.3%) reported as 17,003–20,000 TL, 12 of them (13%) reported as 20,001 TL and 30,000 TL, 15 of them (16%) reported as 30,001–40,000 TL, 22 of them (24%) reported their income in between 40,001 TL and 50,000 TL. Lastly, 21 of them (23%) reported their income as 50,000 TL or higher.

### 2.3. Measures

#### 2.3.1. Measures in the Quantitative Study

Demographic Information Form. A demographic form developed by the first and second authors was utilized to assess the individual and relational characteristics of the participants. The questions about participants’ individual characteristics included gender identity, age, income, and education level. The question about relationships pertained to the duration of participants’ marriages.

Partner Parentification. A single item, developed by three relationship researchers, was used to assess the level of sense of being parentified by a partner, as the literature at the time of development lacked previously verified multi-item scales for measuring partner parentification. The item was: “If you think that you have taken on the role of a parent to your spouse in your marriage, could you please indicate to what extent the parentification you display to your spouse occurs in your marriage?” Partner parentification was rated on a 5-point Likert-type scale, ranging from 1 (never) to 5 (always). Higher scores indicated higher partner parentification, allowing participants to indicate both the presence and absence of perceived parentification. Prior to responding, participants had been provided with a brief formal definition that partner parentification in this context refers to the perception of assuming excessive caregiving, emotional responsibility, decision-making, or regulatory functions toward one’s spouse that are more typically associated with a parental role.

Childhood Parentification. A 22-item subscale of the Parentified Child Scale—Adult Version (PCS–A), developed by Zencir and Haskan Avcı (2019), was utilized to assess general parentification. The sample items are as follows: “When a family member is in trouble, they immediately call me.”, and “Household chores (such as washing dishes, laundry, cleaning, etc.) were my responsibility rather than my mother’s/father’s.” General parentification was rated on a 5-point Likert scale (1 = strongly disagree; 5 = strongly agree). Reverse coding was performed for Items 8, 10, 12, and 22. Higher scores indicated higher parentification. The internal consistency of the subscale, as calculated by Cronbach’s Alpha coefficient, was 0.90.

Couple Burnout. A 10-item Couple Burnout Measure—Short Form (CBMS), developed by Pines et al. (2011) and adapted to Turkish by Çapri (2013), was utilized to assess couple burnout. Specifically, physical and emotional symptoms, as well as mental exhaustion, were measured in the context of couple relationships. The sample item is as follows: “disappointed with partner”. Couple burnout was rated on a 7-point Likert scale (1 = never; 7 = always). High scores indicated higher couple burnout. The internal consistency of the subscale, as calculated by Cronbach’s Alpha coefficient, was 0.94.

#### 2.3.2. Measures in the Qualitative Part

In this study, qualitative data were collected alongside the quantitative data through Google Forms. Following the quantitative assessment tools, participants were given a closed-ended question that simply asked whether they sense they are being parentified in their marriage. Those who said “yes” were given an extra question to describe their experiences of being parentified by their partner in their marriage by asking the following question: “If you feel like your partner parentifies you, can you give examples of these situations? In which areas do you take on a parental role towards your partner?” Written answers from participants were collected and saved verbatim for the analysis.

### 2.4. Data Analysis

#### 2.4.1. Quantitative Part

Before model testing, descriptive statistics and bivariate correlations were analyzed using IBM SPSS Statistics for Windows, Version 29.0 (IBM Corp., Armonk, NY, USA). To evaluate the correlations, Cohen’s (1988) guidelines were followed, where correlations between 0.10 and 0.29 are considered small (weak), between 0.30 and 0.49 are considered medium (moderate), and between 0.50 and 1.00 are considered large (strong). The hypothesized model was tested via path analysis in Jamovi, Version 2.5.6 (The Jamovi Project, 2024).

#### 2.4.2. Qualitative Part

In qualitative data analysis, content analysis was employed, which allows researchers to systematically identify, categorize, and interpret the patterns and themes within the data (Cavanagh, 1997). Specifically, deductive content analysis was employed in the current study because existing theory in the parentification literature supports its application in the parent–child relationship context (Catanzaro, 1988). In the current study, we examined the experiences of parentification among participants in a new context: romantic relationships. Therefore, deductive content analysis presented itself as the best method because it mainly aims to retest the earlier theory in a new or different context (Cavanagh, 1997; Elo & Kyngäs, 2008). Three steps were followed as recommended by Elo and Kyngäs (2008), which are preparing, organizing, and reporting the data. The categories were also evaluated for their distinctiveness and coherence in presenting the data. The qualitative analysis reached data saturation, as all data fit the existing categories well. Two researchers independently coded the data to enhance the credibility and trustworthiness of the analysis (Lincoln & Guba, 1985; Nowell et al., 2017). The intercoder agreement among the researchers was 95%. The categories were not mutually exclusive, allowing for individual responses to be assigned to multiple themes (e.g., both instrumental and emotional parentification) when appropriate. All discrepancies were resolved through discussion, and the process continued until consensus was reached. Additionally, the demographic characteristics of participants and direct quotations were presented in the methods and results sections to address concerns about transferability.

## 3. Results

### 3.1. Quantitative Data

#### Preliminary Analyses

Descriptive statistics, bivariate correlations among the variables, and gender differences for each variable were tested. The variables in the current study were correlated, ranging from small (e.g., between childhood parentification and couple burnout, *r* = 0.22, *p* < 0.001) to medium effect sizes (e.g., between partner parentification and couple burnout, *r* = 0.36, *p* < 0.001). Accordingly, the means, standard deviations, and bivariate correlations are presented in Table 2.

Additionally, the correlations of age and marital duration to relevant variables of the study were calculated. The results revealed that neither age nor marital duration was significantly associated with partner parentification (*r* = 0.00, *p* > 0.05; *r* = 0.05, *p* > 0.05, respectively), childhood parentification (*r* = 0.04, *p* > 0.05; *r* = 0.03, *p* > 0.05, respectively), and couple burnout (*r* = 0.02, *p* > 0.05; *r* = 0.08, *p* > 0.05, respectively). An independent samples t-test was conducted to examine whether there are any gender differences in the relevant variables of the current study. The results indicated that there was not a significant difference between women (*M* = 47.11, *SD* = 16.37) and men (*M* = 47.68, *SD* = 13.27) in their childhood parentification scores: t(251,743) = −0.32, *p* = 0.75. There was not a significant difference between women (*M* = 25.82, *SD* = 13.69) and men (*M* = 23.49, *SD* = 12.84) in their couple burnout scores, as well: t(281) = 1.41, *p* = 0.16. Despite these findings, there was a significant difference between women (*M* = 2.34, *SD* = 1.25) and men (*M* = 1.93, *SD* = 1.31) in their partner parentification scores: t(281) = 2.60, *p* = 0.01.

Mediator Role of Partner Parentification in the Association between Childhood Parentification and Couple Burnout

**Figure 1:**
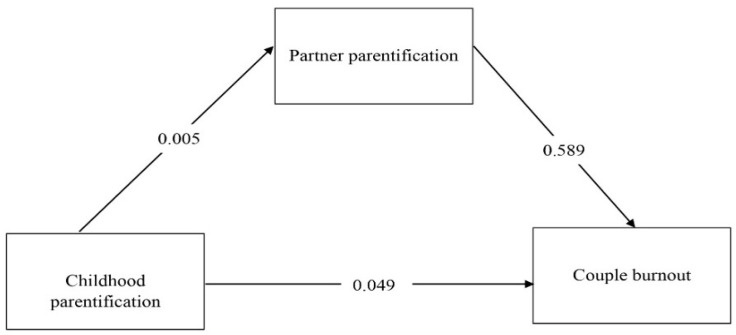
The path model tested in the mediation analysis.

It was hypothesized that the reports of partner parentification would mediate the association between the reports of childhood parentification and couple burnout (Figure 1).

The results of the mediation analyses revealed that the total effect of childhood parentification reports on couple burnout was significant (H1 confirmed; *B* = 0.196, *t* = 3.87, *p* < 0.001). With the inclusion of the mediator (the reports of partner parentification), the impact of the reports of childhood parentification on couple burnout (*B* = 0.129, *t* = 2.62, *p* = 0.009). The indirect effect of the reports of childhood parentification on couple burnout via the reports of partner parentification was significant (H2 confirmed; *β* = 0.066, *t* = 3.32, *p* < 001). 33.9% of the effect of reports of childhood parentification on couple burnout is indirectly mediated by reports of partner parentification; that is, reports of partner parentification partially mediate the association between reports of childhood parentification and couple burnout (see Table 3).

### 3.2. Qualitative Data

According to the existing theoretical framework, two main categories were established: emotional parentification and instrumental parentification (provided as Supplementary Materials). An analysis of the participants’ responses revealed that all responses fell neatly into these predefined categories, making it unnecessary to create an additional category. In conclusion, the researchers determined that all participants’ responses were classified entirely within these established categories.

The majority of participants (*n* = 50) reported experiencing parentification in their relationships with partners, primarily through instrumental means. This included tasks such as cooking, cleaning, shopping for household items, and managing finances. In a similar vein, most participants (*n* = 67) described their experiences of parentification in their partner relationships through emotional means. This involved caring for, guiding, and supporting their partners emotionally, as well as participating in decision-making processes. Detailed explanations of these themes, along with quotations from participants, are provided in Table 4.

## 4. Discussion

The current study aimed to investigate the relationships among reports of childhood parentification, partner parentification, and couple burnout among married participants (for at least 6 months). The results showed a positive relationship between reports of childhood parentification and reports of couple burnout, and that reports of partner parentification partially mediated this relationship. The results are examined below by drawing on the existing literature to enrich our understanding.

The first hypothesis of this study, which posits a positive relationship between reports of childhood parentification and levels of couple burnout, was confirmed, and the results aligned with the existing literature. Previous studies (Baggett et al., 2013; Tolmacz et al., 2025; Zencir, 2021) similarly reported a negative association between the perceived levels of childhood parentification and relationship satisfaction. As discussed by Tolmacz et al. (2025), retrospective accounts of parentification during childhood could lead to lower expectations regarding one’s emotional needs being satisfied by close others, therefore those with higher perceived experiences in childhood parentification might hesitate to or experience difficulty in sharing their needs and wishes, which eventually could lead their needs and wishes being unmet and result in couple burnout. Similarly, as reported by Zencir (2021) and Hooper et al. (2011a, 2011b), being parentified in childhood may endanger one’s emotional well-being and, therefore, constitute a risk factor for emotional exhaustion and burnout.

In addition, as discussed by Baggett et al. (2013), reports of being parentified in childhood may be associated with insecure attachment. Those with higher levels of reported childhood parentification may develop a cognitive schema that leads them to feel valuable only when they serve a purpose. This is also evident in previous research, which demonstrates that elevated levels of childhood parentification may impede an individual’s capacity to develop a positive self-image, as these experiences are associated with diminished self-worth (Black & Sleigh, 2013). Similarly, they may believe they are responsible for the well-being of those around them. Since people with those experiences are unlikely to observe healthy boundaries with themselves and others (Kerig, 2005), they may believe that they would be rejected if they maintain a healthy level of boundaries with close others due to their belief that their partner would not love them unconditionally (also see Hooper, 2007).

Consistent with our expectations, as stated in the second hypothesis of this study, the relationship between reports of childhood parentification and couple burnout was partially mediated by reports of partner parentification. This finding is consistent with the existing literature, which shows a relationship between attachment-related cognitions and childhood parentification (Baggett et al., 2013; Madden & Shaffer, 2016). Attachment theory (Ainsworth, 1989; Bowlby, 1989) emphasizes that the early bond formed with a caregiver lays the foundation for the child’s expectations about what happens to them. This initially relatively primitive system becomes internalized, determining one’s expectations about others. This early bond between caregiver and child provides a framework for shaping later relationships (e.g., with peers and romantic partners) and is referred to as an “internal working model.” Therefore, when children perceive their caregivers as emotionally open and responsive to their needs, the physical environment is perceived as reliable, relationships are seen as enjoyable, and the self is valued. However, in an insensitive environment, both the physical environment and others are perceived as unreliable and untrustworthy, and the child regards oneself as undeserving or unworthy. Consistent with attachment theory, individuals who experienced childhood parentification may be more likely to find themselves in similar cycles, perceiving themselves as caregivers for a dependent other by taking on excessive emotional and instrumental responsibilities (see also Engelhardt, 2012; Hooper, 2007; Tolmacz et al., 2025). Therefore, this situation could exacerbate the effects of the levels of sense of being parentified during childhood on couple burnout.

The current study’s findings additionally indicated a gender-related difference in partner parentification levels, with females exhibiting higher scores than males. This observation is novel and might be interpreted as a cultural nuance in gendered marital role expectations, given that Sakallı Uğurlu et al. (2021) identified stereotypes about married men and women. The results revealed that the most common stereotypes concerning married women aligned with traditional gender roles prevalent in Turkish culture, such as benevolence, affection, and compassion, and with expectations of self-sacrifice, nurturance, housekeeping, domesticity, and family orientation. The same research also uncovered prominent stereotypes of married men, such as being carefree, selfish, or self-indulgent. At the same time, their expected roles included being a family man, household head, and provider. Therefore, women may be more inclined to internalize relational responsibility and emotional caregiving roles within romantic partnerships, thereby increasing their susceptibility to partner-driven parentification dynamics.

In addition, according to Carlson (2022), the most mutually advantageous arrangement for romantic relationships is the one in which both partners share equal responsibility. Not only responsibilities for instrumental duties (e.g., cooking, cleaning, childcare), but also responsibilities for emotional work (i.e., caring, guidance, support) were found to be critical for relationship satisfaction (Pedersen et al., 2011; Sprecher, 1986; Strazdins & Broom, 2004). However, the current literature on partner parentification lacks studies that unveil the experiences of partner parentification; therefore, the qualitative findings are discussed next.

This study also qualitatively collected and analyzed individuals’ perceptions of taking on a parental role toward their partners. The qualitative assessment of partner parentification is novel, as it has not been investigated previously. The results of the qualitative part showed that parentification during childhood by parents and being parentified by a partner in couple relationships are related to taking greater responsibilities in similar domains, which are instrumental (i.e., cooking, cleaning, managing finances) and emotional (i.e., guidance, support, caring) (Hooper, 2007; Jurkovic, 1997). Boszormenyi-Nagy and Spark (1973) defined parentification as the act of placing one’s partner or child in the parental role. This definition emphasizes that one’s partner or child is expected to serve as an advocate, care provider, or protector for the individual. Similarly, an adult places themselves in the child’s position, expecting that another adult will fulfill their needs. Although later definitions (e.g., Chase, 1999; Jurkovic, 1997) primarily focused on the child’s parentification by their parent or parents, the current study highlights that similar domains are also considered or perceived as partner parentification.

## 5. Limitations and Future Directions

Despite the strengths of this study, including its multi-method approach that enables researchers to evaluate a phenomenon both quantitatively and qualitatively, thereby gaining a comprehensive understanding of the relationships among variables under investigation, this study also has several limitations. First, the study sample primarily consisted of women with relatively high educational levels. Dariotis et al. (2023) conducted a systematic review of studies on parentification and found that most participants in those studies were women. Although the overrepresentation of women in parentification research is a well-known limitation, it may still limit the applicability of findings to other populations, such as men or individuals with lower educational levels.

Second, this study employed convenience and snowball sampling methods, which may introduce selection bias and limit the representativeness of the sample and the generalizability of the findings. Another limitation arises from the self-report nature of the study variables, particularly given that recalling childhood parentification may be influenced by recall bias. A further limitation is that partner parentification was measured using a single, newly developed item. Although practical, single-item assessments may be more susceptible to measurement error and warrant confirmation using validated multi-item scales in future research. Furthermore, this study used a correlational, cross-sectional design, which limits the ability to make causal inferences and the researcher’s capacity to observe changes in these relationships over time.

While attachment theory and the fulfillment of personal needs offered the conceptual basis for this research, these factors were not directly measured. The study mainly examined the straightforward descriptive link between childhood parentification and outcomes in romantic relationships. As a result, we did not examine specific mediating factors (e.g., attachment styles or the willingness to express needs) that could clarify how parentification leads to relational issues. Future research should incorporate these variables to deepen understanding of the psychological mechanisms involved. Additionally, data were gathered from only one member of the romantic pair (single-informant approach). While this sheds light on that individual’s view of parentification and relationship quality, it overlooks the dyadic aspect of romantic interactions. Future studies should adopt a dyadic methodology by collecting data from both partners (e.g., utilizing the Actor-Partner Interdependence Model). This approach would enable a more thorough understanding of how one partner’s childhood experiences of parentification may affect the other partner’s relationship outcomes, referring to gender differences, as well.

While couple burnout is often seen as a condition specific to romantic relationships, it can sometimes overlap with general burnout or broader signs of emotional exhaustion (see Pines et al., 2011). Since our data lacked a measure of overall burnout, we were unable to evaluate its unique effects or potential confounding factors. Future studies are suggested to incorporate assessments of both relationship-specific and general burnout to clarify whether parentification uniquely predicts couple-related burnout.

Another significant limitation concerns the collection of qualitative data. In this study, qualitative data were collected through written responses of participants. Therefore, the nature of data collection in the qualitative part could present only a limited in-depth exploration of participants’ experiences. Additionally, most participants who attended the qualitative part were women; therefore, the results are generally limited in terms of generalizability.

Despite these limitations, this study contributes to the literature by exploring partner parentification, which is not under investigation and therefore previously unexplored (to the best of our knowledge). Additionally, this study highlighted its potential role in couple burnout. Future studies are recommended to include diverse samples (i.e., gender, education, culture, dating couples, same-sex couples) and longitudinal designs to capture further insights into relationships among variables. Previous research (e.g., Hooper et al., 2011a; Zencir, 2021) has identified childhood parentification as a risk factor for psychological well-being. However, in the current study, we did not assess psychopathology or personality disorders that may be associated with childhood parentification. Future studies are recommended to incorporate various mental health variables, such as depression, substance use, and personality disorders. In the same vein, we did not differentiate between the types of parentification in the current study (emotional or instrumental); therefore, future studies are recommended to investigate how they contribute to the relationships among variables, either together or separately. Furthermore, some situations (e.g., single parenting, migration, mental health challenges in parents, lower socio-economic status, having a child with a disability or chronic illness, and family conflict) have been discussed as potential risk factors for the parentification of the child (see Dariotis et al., 2023; Kerig, 2005; Masiran et al., 2023; Nuttall et al., 2018); however, the current study did not assess this background information. Future research should consider these factors to better understand the parentification phenomenon and its effects on individuals’ future outcomes.

All in all, these approaches could lead to a deeper understanding of the relationships among the variables that are under investigation in the current study. Additionally, qualitative data collection through various means (e.g., in-depth, face-to-face interviews) could be employed in future studies to gain a deeper understanding of partner parentification. Furthermore, the qualitative assessment of partner parentification experiences could pave the way for the development of reliable and valid assessment tools to measure partner parentification.

## 6. Practical Implications

The findings of this study have significant implications for clinical practice, particularly in addressing egalitarian boundaries in marriage and fostering healthier relationships. The key finding, regarding the positive relationship between reports of childhood parentification and couple burnout, and the partial mediator role of reports of partner parentification, underscores the need for clinical interventions targeting existing cognitions and beliefs regarding roles and responsibilities that prevent individuals from maintaining healthy boundaries.

Burnout can be a challenging experience for individuals and their partners. Assessing childhood parentification experiences during couple therapy may help individuals recognize and process the impact of past family roles and their effects on their current romantic relationships. Also, as discussed in the previous literature (i.e., Madden & Shaffer, 2016; Tolmacz et al., 2025; Zencir, 2021), several key aspects can be addressed while working with individuals with childhood parentification experiences, such as enhancing self-awareness regarding personal needs, expressing or sharing those needs with others, anxious cognitions or beliefs regarding trusting close others concerning their needs would be met in relationships, promote secure attachment patterns by encouraging constructive communication skills, delegating responsibilities to others, and reciprocity of love and affection.

As a result, therapies aimed at reducing couple burnout should consider individuals’ earlier family relationships and parenting experiences. By fostering self-awareness, they can help clients who suffer from an unequal distribution of roles and responsibilities, which risks individual and couple well-being, break free from maladaptive cognitions and schemas that cause problems in their existing romantic relationships. Additionally, therapists can equip couples with the tools they need to establish healthy boundaries and develop emotion regulation skills, thereby reducing exhaustion and couple burnout. These strategies can help individuals and couples achieve long-term relational well-being and satisfaction.

## 7. Conclusions

This study posits that childhood parentification has ongoing effects on adult romantic relationships through its association with partner parentification and couple burnout. Using a multi-method design, we found that partner parentification partially mediates the association between childhood parentification and couple burnout, indicating that early caregiving roles create templates for unequal dynamics in adult partnerships. The qualitative findings further revealed how parentified individuals recreate familiar caregiving patterns with their spouses, often at the expense of their own well-being. These findings highlight the importance of addressing childhood family dynamics in couples therapy. Mental health professionals should assess for parentification histories and help clients recognize how early caregiving roles may sustain maladaptive patterns in current relationships. By fostering self-awareness, promoting healthy boundaries, and challenging cognitions related to responsibility, therapists can help individuals establish more balanced, reciprocal partnerships. Future research should explore the differential impacts of emotional versus instrumental parentification, examine psychopathological factors, and develop validated assessment tools for partner parentification. Ultimately, this research shows that while childhood burdens can cast long shadows over adult relationships, with appropriate therapeutic support, individuals can transform these patterns and create healthier, more sustainable relationships characterized by mutual support and sincere emotional connection.

## Figures and Tables

**Table 1:** Demographic Characteristics of the Participants (*N* = 283).

	*F*	*%*
Gender		
Women	179	63.3
Men	104	36.7
Age		
18–29	80	28.3
30–45	127	44.9
45–65	76	26.9
Duration of Marriage		
6 months–1 year	38	13.4
1 year–5 years	63	22.3
5 years–10 years	32	11.3
10 years–15 years	62	21.9
15 years–20 years	48	17.0
20 years–25 years	22	7.8
Above 25 years	18	6.4
Educational Level		
Primary school	6	2.1
Middle school	3	1.1
High school	23	8.1
Associate’s degree	19	6.7
Undergraduate	161	56.9
Master’s degree	61	21.6
Post-graduate	10	3.5
Income		
Unemployed	37	13.1
Below minimum wage	9	3.2
Minimum wage	8	2.8
17.003–20.000	9	3.2
20.001–30.000	26	9.2
30.001–40.000	32	11.3
40.001–50.000	51	18.0
Over 50.000	111	39.2
Total	283	100

Note. At the time of data collection, the minimum wage in Türkiye was 17,002 TL, and 1 USD was approximately 33 TL.

**Table 2:** Bivariate correlations among study variables.

	1	2	3
1. CB	1	0.224 **	0.357 **
2. CP	0.224 **	1	0.236 **
3. PP	0.357 **	0.236 **	1
*M*	24.96	47.32	2.19
*SD*	13.41	15.28	1.28

Note. CB = couple burnout; CP = childhood parentification; PP = partner parentification, ** *p* < 0.01.

**Table 3:** Mediation analysis.

Total Effect		Direct Effect		Indirect Effect of CP on CB					
Coefficient	*p*	Coefficient	*p*		Coefficient	SE	t	*p*	CI %95
0.196	0.000	0.129	0.009	H2: CP->PP->CB	0.066	0.020	3.32	0.000	0.001; 0.029

Note. CP = childhood parentification; PP = partner parentification; CB = couple burnout.

**Table 4:** Themes and Representative Quotations of Parentification in Partner Relationships.

Theme	Description	Representative Quotation
Instrumental Parentification	Participants described taking responsibility for practical and household-related tasks within their partner relationships, including domestic chores, childcare, and financial management.	“I cook the meals and do most of the house cleaning.” (P18) “When my husband is tired and stressed after going to work, I take on the chores at home even though I am tired too.” (P36) “Managing finances… I feel like he can’t do the housework, I do it.” (P47)
Emotional Parentification	Participants described assuming emotional responsibility for their partners, including providing emotional caregiving, guidance, reassurance, and involvement in decision-making.	“Being the one who is always strict with the children, being the one who is always consoling him, being the one who always thinks of everything, just so that he doesn’t get upset.” (P3) “Considering his hunger/fullness and taking action.” (P9) “Telling him that he is a very valuable person when he feels worthless.” (P11) “I have to remind him every time about what needs to be done. If I have to give the simplest example, when he is going out of town. On the weekends, train tickets are usually sold out immediately. I have to remind him every time to get them on time. When I don’t remind him, the tickets are sold out, and he has to take the bus, even though he does not like it at all. He wants me to plan all the things we will do together. When I ask for advice, he says, “It’s up to you,” but he doesn’t act without getting my advice.” (P91)

## Data Availability

Data will be provided upon reasonable request.

## Supplementary Materials

The following supporting information can be downloaded at: https://www.mdpi.com/article/10.3390/bs16020177/s1. Responses to qualitative questions and their coding and relevant themes can be found in the document.
